# Pharmacokinetics, safety, and pharmacodynamics of intravenous methoxyethyl etomidate hydrochloride (ET-26-HCl) in Chinese participants with hepatic impairment

**DOI:** 10.3389/fphar.2026.1827752

**Published:** 2026-07-30

**Authors:** Yue Hu, Wenbo Zheng, Hong Zhang, Lize Li, Lei Diao, Xiaoran Yang, Yanhua Ding

**Affiliations:** 1 Phase I Clinical Research Center, First Hospital of Jilin University, Changchun, China; 2 Avanc Pharmaceutical Co., Ltd., Jinzhou, China; 3 Shanghai Fosun Pharmaceutical Group Co., Ltd., Shanghai, China

**Keywords:** ET-26-HCl, hepatic impairment, pharmacodynamics, pharmacokinetics, safety

## Abstract

**Background:**

Intravenous methoxyethyl etomidate hydrochloride (ET-26-HCl) is a novel etomidate analogue. We aimed to investigate the pharmacokinetics, safety and pharmacodynamics of ET-26-HCl in individuals with hepatic impairment.

**Methods:**

In this open-label, parallel study, we compared the pharmacokinetics, pharmacodynamics and safety of a single intravenous bolus dose of 0.8 mg kg^-1^ ET-26-HCl for 1 min between patients with mild or moderate hepatic impairment (Child‒Pugh A/B) and those of healthy participants matched for sex, age, and body weight. Each cohort included eight participants. The pharmacokinetics of ET-26-HCl and its predominant metabolite, ET-26-acid, were characterized. Safety was assessed through vital signs, laboratory tests, physical examinations and adverse events (AEs). Pharmacodynamics were evaluated using the Modified Observer’s Assessment of Alertness/Sedation (MOAA/S) and the bispectral index (BIS).

**Results:**

Twenty-four participants completed the study. Compared with that of healthy participants, the arterial plasma AUC geometric means of ET-26-HCl were similar in patients with a moderate impairment and increased in those with mild impairment. The geometric mean ratios for the venous plasma AUC of ET-26-HCl were ∼1.31- and ∼1.48-fold greater in patients with mild and moderate impairment, respectively, than in healthy participants. Most AEs were mild to moderate. No serious AEs occurred. The degree of hepatic impairment had little effect on the MOAA/S and BIS of ET-26-HCl.

**Conclusion:**

Venous plasma ET-26-HCl exposure was ∼1.31- and ∼1.48-fold greater in patients with mild and moderate hepatic impairment, respectively, than in matched healthy controls. ET-26-HCl was generally safe and well tolerated by all participants in this study. Importantly, these findings should not be extrapolated to patients with severe hepatic impairment.

**Clinical Trial Registration:**

NCT06176716, https://clinicaltrials.gov/

## Introduction

Etomidate, a γ-aminobutyric acid type A receptor agonist, is a hypnotic that is used to induce anaesthesia and is well known for its remarkably stable cardiorespiratory profile (Giese and Stanley, 1983; Forman, 2011; Valk and Struys, 2021). However, the use of etomidate is limited because the prolonged suppression of adrenocortical steroid synthesis can increase mortality in critically ill patients (Valk and Struys, 2021; Wagner et al., 1984; Hildreth et al., 2008; Cotton et al., 2008). Over the past few decades, various analogues of etomidate, such as methoxycarbonyl-etomidate, carboetomidate, and ABP-700, have been developed to reduce side effects, but a few concerns persist (Cotten et al., 2009; Cotten et al., 2010; Struys et al., 2017).

Intravenous methoxyethyl etomidate hydrochloride (ET-26-HCl), a novel analogue of etomidate synthesized by altering its ester side chain, was developed by Avanc Pharmaceutical Co., Ltd. The results of preclinical and clinical studies (phases I to II) have suggested that this modification reduced adrenal suppression while maintaining the advantages of etomidate (Wang et al., 2017a; Wang et al., 2017b; Zhang et al., 2020; Zhang et al., 2021; Chang et al., 2022; Yin et al., 2025). Moreover, previous studies have shown that the anaesthetic potency of ET-26-HCl is similar to that of etomidate and that it results in faster spatial orientation recovery from anaesthesia than does propofol (Yin et al., 2025; Wang et al., 2017c).

Previous studies have shown that both ET-26-HCl and its main inactive hydroxylated metabolite, ET-26-acid (ETA), are widely and rapidly distributed in the liver after intravenous administration. The plasma protein binding rate of ET-26-HCl ranges from 62.7% to 94.4% in different species (Zhang et al., 2019; Yu et al., 2020). Similar to other general anaesthetics, ET-26-HCl is extensively metabolized by cytochrome P450 (CYP) enzymes (Restrepo et al., 2009). The results of *in vitro* studies have suggested that CYP2C19 primarily mediates ET-26-HCl metabolism. A radiolabelled mass balance study provided clinical evidence of ET-26-HCl metabolism and indicated that liver metabolism is the main pathway of ET-26-HCl clearance.

Hepatic impairment may modify hepatic blood flow, plasma protein binding, and biliary excretion, thereby altering drug metabolism and reducing drug elimination. The pharmacokinetics (PK) of ET-26-HCl may be altered in patients with impaired liver function. Therefore, clarifying the impact of hepatic impairment on the pharmacokinetics of ET-26-HCl is important for guiding labelling and drug dosage adjustments for patients with liver dysfunction (Rodighiero, 1999; Delcò et al., 2005; Verbeeck, 2008; Pena et al., 2016.; Cobbina and Akhlaghi, 2017).

In accordance with the recommendations of the Chinese National Medical Products Administration (NMPA), the Food and Drug Administration (FDA), and the European Medicines Evaluation Agency (EMEA) for the effects of liver disease on the pharmacokinetics of drugs, in this study, we evaluated the PK profile, safety, and pharmacodynamics (PD) of ET-26-HCl in participants with mild or moderate hepatic impairment compared with healthy controls (US Food and Drug Administration, 2003; EMEA, 2005; China Food and Drug Administration, 2012).

## Methods

### Ethics statement and registration

This clinical study was conducted at the First Hospital of Jilin University, Changchun, China. The trial was approved by the Institutional Research Ethics Committee of the hospital (approval number: 23Y316-001). All procedures were performed in accordance with the principles of the Declaration of Helsinki and Good Clinical Practice standards. This trial was initiated in November 2023 and ended in January 2024. All the participants signed informed consent forms before participating in the trial. The study was registered with ClinicalTrials.gov (Registration No. NCT06176716, https://clinicaltrials.gov/).

### Study design

This study was designed as a single-centre open-label, parallel-group study of ET-26-HCl. According to the results of previous phase I–II studies of ET-26-HCl that are currently available, the recommended dose for unconsciousness is 0.8 mg kg^-1^, which is safe and effective (Yin et al., 2025). In addition, the sample size of the study was designed for estimation rather than hypothesis testing. In accordance with the guidelines of the Chinese NMPA, FDA and EMEA for the sample size of the pharmacokinetics study, a total of 8–12 participants were sufficient to meet the needs of the evaluation of the pharmacokinetic characteristics. Eight participants with normal hepatic function, 8 participants with mild hepatic impairment (Child‒Pugh A) and 8 participants with moderate hepatic impairment (Child‒Pugh B) were included. All participants received an intravenous (IV) bolus dose of 0.8 mg kg^-1^ ET-26-HCl for 1 min.

The participants were admitted to the study site the day before the study drug was administered (day 1) and were required to fast for a minimum of 8 h overnight before the study drug was administered. Fasting was a compulsory preoperative safety requirement inherent to all investigational anesthetic agents. Water was not permitted for 2 h before dosing. Before dosing, all participants were transferred to a dedicated treatment room equipped with monitoring equipment, emergency equipment and drugs to treat adverse events (AEs). A certified anaesthesiologist was present during drug administration until the participants recovered consciousness and had normal cardiovascular and respiratory function. Additional oxygen (2–10 L min^-1^) was delivered via a face mask during drug administration. During the study period, vital signs and electrocardiograms were used to monitor safety. The bispectral index (BIS) and Modified Observer’s Assessment of Alertness/Sedation (MOAA/S) score were assessed. The participants discharged on day 2 after the safety assessments were performed.

### Participants

In this phase 1 study, eligible men and women aged 18–70 years with a body mass index of 18–32 kg m^-2^ were included. The participants with hepatic impairment had a confirmed clinical diagnosis of either mild (Child–Pugh A) or moderate hepatic impairment (Child–Pugh B) resulting from previous primary liver disease, including nonalcoholic steatohepatitis and viral hepatitis (hepatitis B or hepatitis C). Patients with liver failure, cirrhosis complicated by hepatic encephalopathy, hepatocellular carcinoma (except for the Barcelona stage 0), oesophageal or ruptured gastric varices or bleeding, or severe medical history were excluded. According to the screening, the participants with normal liver function had no previous primary diseases of major organs, no clinically significant abnormal findings on physical examination or in their medical history, and no abnormal clinical laboratory test results. The female participants were not pregnant, were nonlactating, and were using medically acceptable forms of birth control. In addition, the participants were not at risk of having a difficult airway (modified Mallampati score I or II). The mean body weight and age of participants in the normal hepatic function group were within ±10 kg and ±10 years, respectively, of the mean body weight and age of the participants with hepatic impairment. Attempts were also made to ensure comparable male‒female ratios among the groups.

### PK analysis

Arterial and venous PK samples were collected at 0 h (predose), at the end of the dose infusion (1 min after the start of infusion), and 1 min, 3 min, 5 min, 15 min, and 1 h after the infusion stopped, and venous blood samples were also collected at 10 min, 30 min, 2 h, 4 h, 8 h, and 24 h after the infusion stopped. In addition, extra venous blood was collected 1 min and 4 h after the infusion stopped and was used to determine the plasma protein binding. Blood samples were collected into K_2_-EDTA-containing tubes and centrifuged at 1700 g and 4 °C for 10 min, after which the plasma was separated and stored in polypropylene tubes in two equal aliquots at −60 °C to −90 °C until analysis.

The plasma samples were analysed to determine ET-26-HCl and ETA levels using a validated analytical method based on liquid chromatography‒tandem mass spectrometry (LC‒MS/MS) in accordance with the validation principles defined in ICH M10. Protein binding was assessed using equilibrium dialysis. The concentration ranges of the standard curves for the detection of ET-26-HCl and ETA in plasma were 10.0–4000 ng mL^-1^ and 2.00–800 ng mL^-1^, respectively. For the ET-26-HCl and ETA determinations, the accuracy values were −3.25%–2.50% and −4.25%–3.75%, respectively, and the precision values were within 2.60% CV and 8.83% of the CV, respectively. The calibration ranges of the assays for the ET-26-HCl and ETA plasma protein binding determination were 4.00–1,600 ng mL^-1^ and 0.500–200 ng mL^-1^, respectively. With respect to the determination of the plasma protein binding of ET-26-HCl and ETA, the accuracy ranged from −0.83%–3.33% and −1.90%–6.00%, respectively, and the precisions was within 2.49% CV and 5.58% CV, respectively.

### Safety

Safety and tolerability were evaluated according to the National Cancer Institute Common Terminology Criteria for the Classification of Adverse Events (NCI-CTCAE, 5.0) after participants were administered the study drug. Safety was assessed according to the following parameters: adverse events, vital signs, including systolic and diastolic blood pressure, mean arterial blood pressure, heart rate, respiration rate and body temperature, oximetry, continuous 3-lead cardiac monitoring, 12-lead electrocardiograms, physical examination, and laboratory tests.

### Efficacy

During the study, the clinical hypnotic–anaesthetic effect of ET-26-HCl was evaluated by BIS monitoring (BIS VISTA™ monitor; Aspect Medical Systems, Norwood, MA, United States) and MOAA/S score assessments. The BIS scores were recorded every minute until the patients were fully alert after dosing. The MOAA/S scores were recorded before the infusion, at the end of the dose infusion, and every 2 min after the infusion stopped until full recovery. The subjects were considered to have recovered when three consecutive MOAA/S scores of 5 were obtained.

### Statistics

Statistical analyses were performed using SAS 9.4 (SAS Institute, Cary, NC, United States). Descriptive statistics were used to present the PK and PD parameters. The PK parameters were calculated using a noncompartmental model (Phoenix WinNonlin ver. 8.2; Certara, Princeton, United States), as follows: time to maximum observed plasma concentration (T_max_); maximum observed plasma concentration (C_max_); area under the concentration‒time curve from the time of dosing to the last time point with measurable plasma concentrations of drug (AUC_0-t_); AUC from the time of dosing extrapolated to infinity (AUC 0−∞ ); terminal elimination half-life of the drug in plasma (t_1/2_); total clearance adjusted by weight (CL); distribution volume adjusted by weight (V_d_); mean retention time (MRT); free fraction (fu); and terminal phase elimination rate constant (λ_z_). The results are presented as the means (coefficients of variation or standard deviations). The peak time is presented as the median (range). Analysis of variance (ANOVA) was applied to the log-transformed C_max_, AUC_0-t_, and AUC 0−∞ ; the results were back-transformed to obtain the geometric mean ratio (GMR) and 90% confidence interval (CI) [(hepatic impairment)/(healthy control)]. Nonparametric analyses of the time to the BIS peak (T_BISpeak_) were performed using the Wilcoxon rank-sum test for comparisons between patients with impaired hepatic function and matched healthy controls. A simple linear regression model was used for to analyze the relationship between ET-26-HCl exposure and the Child-Pugh score. Scatter plots were generated for ET-26-HCl exposure with respect to the time to full alertness and BIS parameters.

After intravenous bolus injection, ET-26-HCl distributes to the central nervous system via arterial circulation and elicits its pharmacological actions primarily as the unmetabolized parent compound. For this reason, all subsequent PK analyses were primarily based on arterial plasma exposure data of ET-26-HCl, while venous plasma exposure data served as auxiliary evidence. Meanwhile, PD and safety data obtained in this study will be intergrated for overall analysis.

## Results

### Subjects

In all, 53 participants were screened, and of these, 24 participants [8 participants with normal hepatic function, 8 with mild hepatic impairment (Child‒Pugh A), and 8 with moderate hepatic impairment (Child‒Pugh B)] were enrolled and completed the study. The demographic and baseline characteristics of the participants are summarized in Table 1.

**TABLE 1:** Demographic and baseline characteristics of the participants.

Variable	Mild hepatic impairment (N = 8)	Moderate hepatic impairment (N = 8)	Normal hepatic function (N = 8)	Total (N = 24)
Age (years), mean ± SD (range)	51.3 ± 7.19 (43–66)	49.3 ± 10.46 (32–62)	47.6 ± 3.16 (43–52)	49.4 ± 7.37 (32–66)
Height, cm, mean ± SD (range)	163.89 ± 6.416 (155.1–172.6)	163.20 ± 7.059 (155.3–176.1)	166.69 ± 6.274 (156.5–173.1)	164.59 ± 6.485 (155.1–176.1)
Weight, kg, mean ± SD (range)	70.34 ± 8.161 (56.6–78.3)	66.73 ± 11.209 (53.0–84.4)	64.44 ± 2.539 (61.2–68.4)	67.17 ± 8.163 (53.0–84.4)
Body mass index, kg/m^2^, mean ± SD (range)	26.1 ± 3.14 (22–31)	24.9 ± 3.27 (19–29)	23.4 ± 2.62 (21–28)	24.8 ± 3.11 (19–31)
Race, n (%), asian	8 (100)	8 (100)	8 (100)	24 (100)
Sex, n (%)				
Male	7 (87.5)	5 (62.5)	6 (75.0)	18 (75.0)
Female	1 (12.5)	3 (37.5)	2 (25.0)	6 (25.0)
Airway evaluation, n (%)				
I	1 (12.5)	1 (12.5)	5 (62.5)	7 (29.2)
II	7 (87.5)	7 (87.5)	3 (37.5)	17 (70.8)
Allen’s test, n (%)				
Negative	8 (100)	8 (100)	8 (100)	24 (100)
Positive	0	0	0	0
Child–Pugh score, mean ± SD (range)	5.25 ± 0.433 (5–6)	7.38 ± 0.484 (7–8)	NA	NA

SD, standard deviation.

### Pharmacokinetics properties

**FIGURE 1:**
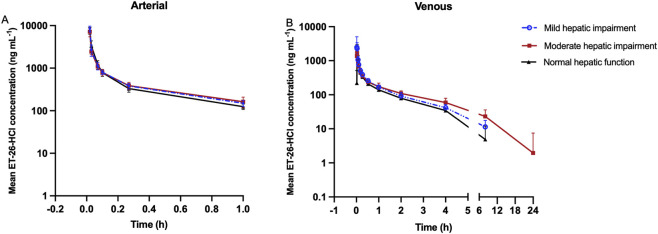
Plasma concentration‒time curves for ET-26-HCl on semilog scales. Left panel, arterial plasma graph **(A)**; Right panel, venous plasma graph **(B)**. The data are presented as the means ± standard deviations. Below the lower limit of quantification (LLOQ) detected either prior to or following T_max_ were assigned a value of zero, whereas Below LLOQ situated between two consecutive quantifiable concentration data points were treated as missing values.

**FIGURE 2:**
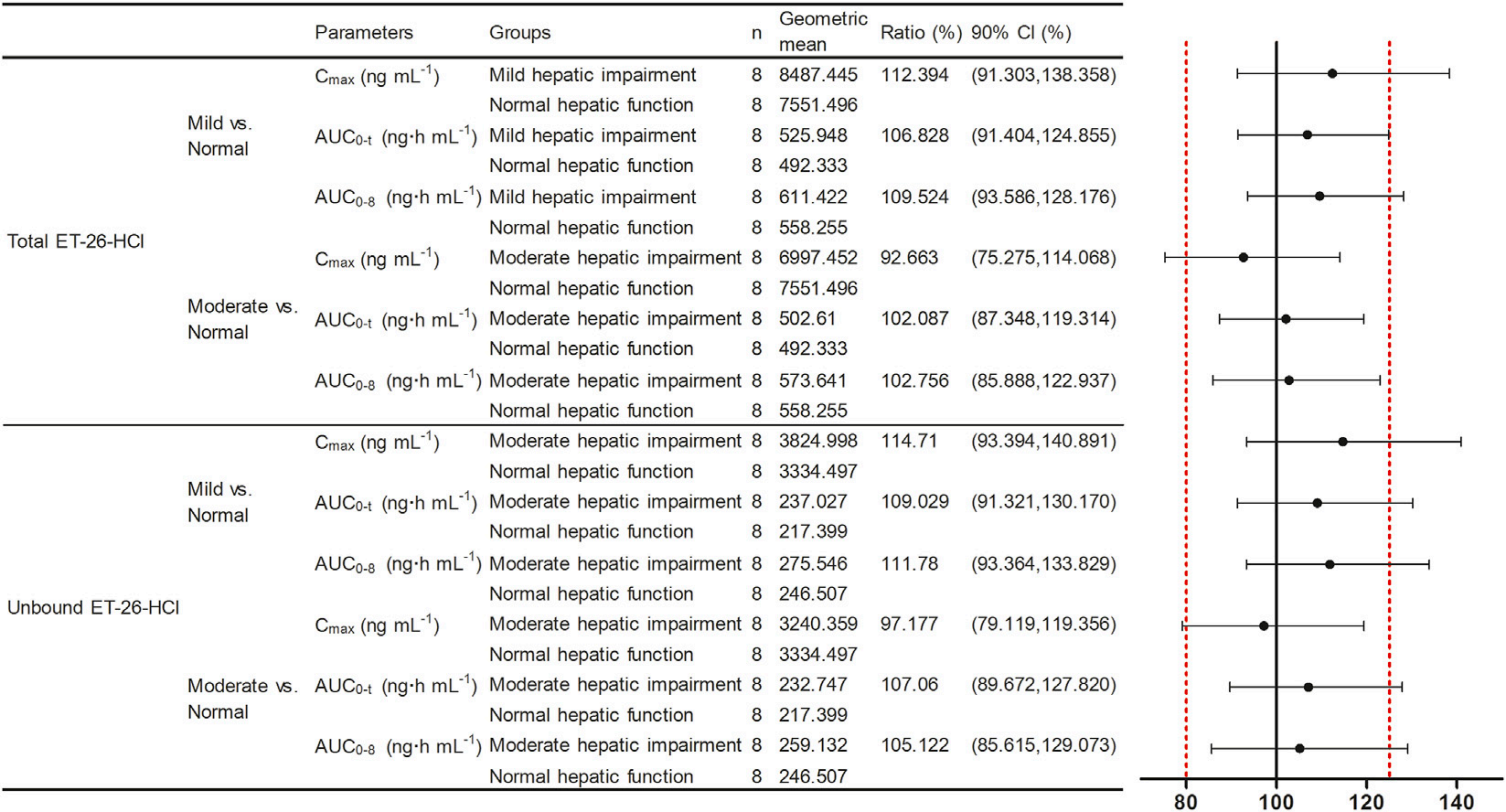
Forest plot of geometric mean ratios (GMRs) and 90% confidence intervals (CIs) for key pharmacokinetic parameters (C_max_ and AUC) of arterial plasma total and unbound ET-26-HCl in patients with mild or moderate hepatic impairment relative to those with normal hepatic function. Normal hepatic function group served as the reference comparator. n = 8 participants per group. Horizontal lines represent 90% CIs; the vertical red lines mark the 80%–125% interval.

**FIGURE 3:**
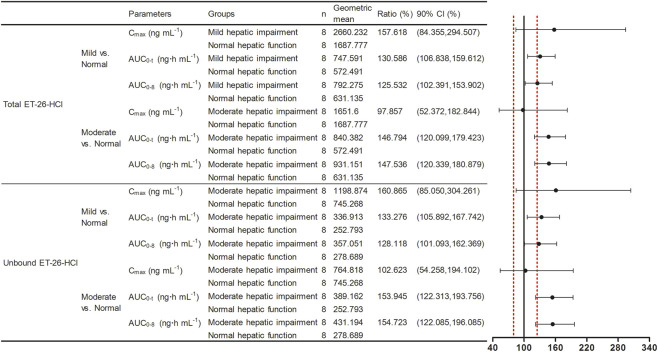
Forest plot of geometric mean ratios (GMRs) and 90% confidence intervals (CIs) for key pharmacokinetic parameters (C_max_ and AUC) of venous plasma total and unbound ET-26-HCl in patients with mild or moderate hepatic impairment relative to those with normal hepatic function. Normal hepatic function group served as the reference comparator. n = 8 participants per group. Horizontal lines represent 90% CIs; the vertical red lines mark the 80%–125% interval.

The mean plasma concentration‒time profiles for ET-26-HCl in the different groups are shown in Figure 1, and the PK parameters are shown in Tables 2, 3. The impact of the hepatic impairment on ET-26-HCl exposure is depicted in Figures 2, 3.

**TABLE 2:** Pharmacokinetic characteristics of total and unbound ET-26-HCl in arterial plasma.

Analyte	Parameters	Mild hepatic impairment (N = 8)	Moderate hepatic impairment (N = 8)	Normal hepatic function (N = 8)
Total ET-26-HCl	C_max_ (ng mL^-1^)	8,490 (13.99)	7,000 (25.77)	7,550 (21.01)
	AUC_0-t_ (ng·h mL^-1^)	526 (11.45)	503 (16.14)	492 (18.22)
	AUC 0−∞ (ng·h mL^-1^)	611 (12.32)	601 (19.06)	558 (17.81)
	λ_z_ (1 h^-1^)	1.77 (13.35)	1.67 (17.95)	1.88 (6.83)
	T_max_ (min)	1.00 (1.00.1.03)	1.00 (1.00.1.00)	1.00 (1.00.1.00)
	t_1/2_ (h)	0.392 (13.35)	0.416 (17.95)	0.368 (6.83)
	CL (L h^-1^)	89.9 (13.59)	87.3 (22.08)	91.6 (15.32)
	V_d_ (L)	50.9 (15.40)	52.4 (28.13)	48.6 (15.11)
	MRT_0-t_ (h)	0.25 (7.80)	0.27 (10.07)	0.23 (3.33)
	${mrt}_{0-\infty }$ (h)	0.44 (14.75)	0.49 (21.10)	0.39 (3.37)
	AUC__%Extrap_ (%)	13.68 (20.51)	15.69 (30.37)	11.79 (6.27)
Unbound ET-26-HCl	C_max,u_ (ng mL^-1^)	3,820 (13.74)	3,240 (26.79)	3,330 (19.18)
	AUC_0-t,u_ (ng·h mL^-1^)	237 (13.40)	233 (21.17)	217 (17.63)
	AUC 0−∞ _,u_ (ng·h mL^-1^)	276 (14.66)	278 (24.78)	247 (17.17)
	CLu (L/h)	40.5 (14.40)	40.4 (20.65)	40.5 (18.47)
	V_d,u_ (L)	22.9 (17.60)	24.3 (30.12)	21.5 (18.33)
	Fu (%)	45.12 (5.18)	46.44 (8.02)	44.25 (6.95)

The data are presented as the geometric means (CV%) except for T_max,_ which is presented as the median (range). C_max_: maximum observed concentration; AUC_0-t_: area under the curve from zero to last time of quantifiable concentration; AUC 0−∞ : area under the curve from zero to infinity; t_1/2_: terminal elimination half-life; T_max_: time to maximum concentration; CL: total clearance; V_d_: distribution volume; MRT: mean residence time; fu: free fraction.

**TABLE 3:** Pharmacokinetic characteristics of total and unbound ET-26 in venous plasma.

Analyte	Parameters	Mild hepatic impairment (N = 8)	Moderate hepatic impairment (N = 8)	Normal hepatic function (N = 8)
Total ET-26-HCl	C_max_ (ng mL^-1^)	2,660 (73.58)	1,650 (73.93)	1,690 (56.73)
	AUC_0-t_ (ng·h mL^-1^)	748 (16.23)	840 (25.74)	572 (16.90)
	AUC 0−∞ (ng·h mL^-1^)	792 (16.48)	931 (27.47)	631 (15.04)
	λ_z_ (1/h)	0.339 (18.21)	0.250 (54.51)	0.406 (23.39)
	T_max_ (min)	1.00 (1.00.2.00)	2.00 (1.00.6.00)	2.00 (2.00.2.00)
	t_1/2_ (h)	2.05 (18.21)	2.77 (54.51)	1.71 (23.39)
	CL (L/h)	69.4 (15.46)	56.4 (21.45)	81.1 (12.52)
	V_d_ (L)	205 (17.08)	225 (38.47)	200 (14.53)
	MRT_0-t_ (h)	1.50 (25.54)	2.13 (60.63)	1.25 (30.86)
	${mrt}_{0-\infty }$ (h)	2.00 (16.72)	3.12 (63.02)	1.83 (22.08)
	AUC__%Extrap_ (%)	5.30 (36.63)	9.44 (28.19)	8.69 (41.96)
Unbound ET-26-HCl	C_max,u_ (ng mL^-1^)	1,200 (76.37)	765 (70.78)	745 (62.23)
	AUC_0-t,u_ (ng·h mL^-1^)	337 (18.71)	389 (32.09)	253 (15.24)
	AUC 0−∞ _,u_ (ng·h mL^-1^)	357 (19.12)	431 (34.04)	279 (13.95)
	CLu (L/h)	31.3 (15.23)	26.1 (16.29)	35.8 (16.50)
	V_d,u_ (L)	92.3 (17.09)	104 (41.63)	88.2 (9.58)
	Fu (%)	45.12 (5.18)	46.44 (8.02)	44.25 (6.95)

The data are presented as the geometric means (CV%) except for T_max,_ which is presented as the median (range), and fu, which is presented as the mean (SD). C_max_: maximum observed concentration; AUC_0-t_: area under the curve from zero to last time of quantifiable concentration; AUC 0−∞ : area under the curve from zero to infinity; t_1/2_: terminal elimination half-life; T_max_: time to maximum concentration; CL: total clearance; V_d_: distribution volume; MRT: mean residence time; fu: free fraction.

After intravenous single-dose infusion of 0.8 mg kg^-1^, the C_max_ of ET-26-HCl was reached within 1–2 min at the end of infusion, as expected. In arterial plasma, the AUC_0-t_ and AUC 0−∞ geometric means of the total ET-26-HCl levels in the hepatic impairment groups were 2.1%–9.5% greater than those in the normal group. The mean t_1/2_ values of ET-26-HCl were similar across the three groups. In venous plasma, the AUC_0-t_ and AUC 0−∞ geometric mean of total ET-26-HCl increased gradually with decreasing hepatic function. The mean t_1/2_ tended to increase. The trends of unbound ET-26-HCl pharmacokinetic parameters in arterial plasma and venous plasma were consistent with those of total ET-26-HCl. In all the groups, the mean Fu values of ET-26-HCl ranged from 44.25% to 46.44%.

The major PK parameters of the predominant metabolite, ETA, in arterial and venous plasma differed among different populations with hepatic impairment (Supplementary information; Supplementary Table S1; Supplementary Figure S1).

The GMRs and 90% CIs of the C_max_ and AUC are shown in Figure 2. In arterial plasma, the 90% CIs of the ratios for C_max_ and AUC of total ET-26-HCl were almost within the 80%–125% interval, but for unbound ET-26-HCl in plasma, those values overlapped with the 80%–125% interval, with the upper limit slightly above 125%. In venous plasma, the GMRs of the AUCs for total and unbound ET-26-HCl in both the mild and moderate hepatic impairment groups were greater than 1.

### Safety and tolerability

ET-26-HCl was generally safe and well tolerated by all participants. No Grade 4 or above AEs were observed in any participant, and no severe AEs occurred. No participants withdrew from the trial because of AEs.

Seventeen participants (70.8%) experienced a total of 36 AEs, 30 of which were adverse drug reactions (ADRs). All the AEs were mild to moderate in severity, except for a Grade 3 AE in which the platelet count decreased in the moderate hepatic impairment group. Owing to changes in severity, this event was reported as an AE, but the value did not significantly change from baseline. This AE occurred after exit examination (day11). Because this AE was considered related to underlying liver disease, it may not be related to the study drug.

The most common ADRs reported in this study were cough (16.7%), elevated blood pressure (16.7%), elevated blood bilirubin (12.5%), and involuntary muscle movement (12.5%). Sedation-related AEs, which were all grade 1 in severity and included bradycardia and respiratory depression, were reported in 3 participants (12.5%), all of whom were in the normal hepatic function group, and the participants recovered without treatment. These data are presented in Supplementary Material; Supplementary Table S2.

### Clinical effects and pharmacodynamics

**FIGURE 4:**
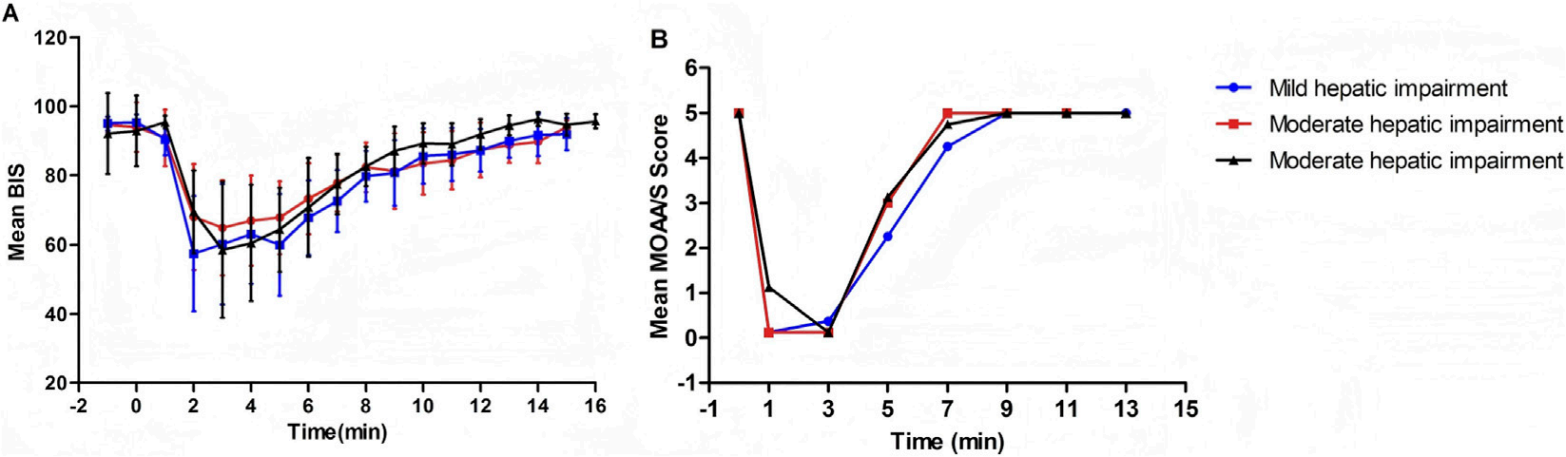
The mean BIS-time curves **(A)** and MOAA/S score-time curves **(B)** for the normal hepatic function, mild hepatic impairment and moderate hepatic impairment groups (the data are presented as the means ± standard deviations).

The observed mean BIS and MOAA/S score–time curves for each group are shown in Figure 4.

All the subjects had a MOAA/S score of 5 at baseline. The time to full alertness was the time measured from the dosing stop time to the attainment of 3 consecutive MOAA/S scores of 5. The times to full alertness ranged from 8.00 min to 12.08 min.

The mean BIS_peak_ values were 61.2, 47.2 and 48.8 for the mild, moderate and normal groups, respectively, and the median T_BISpeak_ values were 4 min in all groups. The mean BIS AUC_0-t_ values were 1,100, 1,080, and 1,300 for the mild, moderate and normal groups, respectively (Supplementary Material; Supplementary Table S3). The GMRs for the BIS-related parameters are shown in Supplementary Material; Supplementary Figure S2.

### Exploratory analysis

The correlation scatterplots of the ET-26-HCl exposure level (AUC 0−∞ ) with the time to full alertness, BIS AUC_0-t_, BIS_peak_ and T_BISpeak_ for each group are shown in Supplementary Material; Supplementary Figures S3-S4. An exploratory analysis of CYP2C19 gene subtypes was conducted, and detailed results are presented in the Supplementary Material; Supplementary Tables S4-S5. The relationship of ET-26-HCl C_max_ or the AUC 0−∞ with the Child-Pugh score, are shown in Supplementary Material; Supplementary Figure S5.

## Discussion

The liver is a pivotal organ governing overall drug pharmacokinetics, and hepatic impairment may modify drug disposition through multiple biological mechanisms (Rodighiero, 1999; Delcò et al., 2005; Verbeeck, 2008). Previous studies have revealed that (Zhang et al., 2019; Yang et al., 2017) hepatic clearance is the major elimination pathway for ET-26-HCl, which is metabolized by CYP2C19. Consequently, impaired liver function may alter the PK profile of ET-26-HCl (Verbeeck, 2008). To our knowledge, the present study is the first to comprehensively characterize the PK, PD (including MOAA/S and BIS) profiles and safety profile of ET-26-HCl in Chinese patients with mild to moderate hepatic impairment.

In this study, no apparent differences in the AUCs of total and unbound ET-26-HCl were observed in arterial plasma between the patients with hepatic impairment and participants with normal liver function. The venous plasma AUCs of total and unbound ET-26-HCl increased progressively alongside the worsening of hepatic function: these values rose by ∼30.6% in patients with mild hepatic impairment and ∼47.5% in those with moderate hepatic impairment. The C_max_ of ET-26-HCl in both arterial and venous specimens was comparable between the moderate hepatic impairment group and the normal hepatic function group, yet it was lower than that measured in the mild impairment group. Of note, venous drug concentrations exhibited substantial interindividual variability (>56.73%). Moreover, the unbound fraction of ET-26-HCl was similar among the three groups. And the CL of ET-26-HCl tended to decrease, whereas the mean t_1/2_ tended to increase. Published evidence (Verbeeck, 2008; Cobbina and Akhlaghi, 2017) has demonstrated that CYP enzyme activity is suppressed to varying extents in patients with liver diseases. Accordingly, the increased exposure to ET-26-HCl and decrease in CL observed in hepatic dysfunction patients are likely attributable to altered metabolic enzyme activity. Furthermore, venous plasma AUC of ETA was greater in patients with hepatic impairment relative to those with normal liver function, and this elevation showed no correlation with the severity of liver injury. As ETA, the primary metabolite of ET-26-HCl, is inactive (Restrepo et al., 2009), the above elevation is clinically irrelevant. The T_max_ of ETA was delayed as liver function deteriorated, suggesting that hepatic dysfunction may slow the hydrolytic metabolism of ET-26-HCl to generate ETA.

Overall, a single intravenous bolus of 0.8 mg/kg ET-26-HCl was well tolerated and safe for all the participants. AEs were more frequently observed in patients with hepatic impairment than in healthy participants (75.0% versus 62.5%). Most reported AEs were classified as mild or moderate and resolved without intervention. The AEs observed in this study was consistent with findings from previous phase I-II studies of ET-26-HCl and other analog anesthetics (e.g., ABP-700) (Struys et al., 2017; Yin et al., 2025; Valk et al., 2018). The most commonly reported AEs in this study were cough, elevated blood pressure, elevated blood bilirubin, and involuntary muscle movement. Although elevated blood pressure was exclusively observed in patients with mild hepatic impairment, these patients presented unstable baseline blood pressure. Involuntary muscle movement was reported in the moderate hepatic impairment group, but it was transient and relieved without treatment. In addition, ET-26-HCl injection had a small effect on the respiratory system in patients with hepatic impairment. The incidence and severity of myoclonus were comparable between the hepatic impairment group and the normal hepatic function group.

In patients with liver cirrhosis, central nervous system-active agents may trigger hepatic encephalopathy and induce prolonged drug effects (Suh et al., 2014). The influence of hepatic function on the PK of propofol has been well characterized in prior studies (Servin et al., 1988; Servin et al., 1990). Nevertheless, no relevant studies have been performed on etomidate or its structural analogues to date. In this study, we first report the results of etomidate analogues in patients with hepatic impairment. Although the venous plasma AUC of ET-26-HCl slightly increased gradually with decreasing hepatic function, the degree of hepatic impairment had minimal effect on the PD (MOAA/S, BIS) profile of ET-26-HCl. The hypnotic profile was consistent with that of previous studies (Zhang et al., 2021; Wang et al., 2017c). Importantly, no cases of hepatic encephalopathy were identified throughout the trial.

According to the drug label of etomidate, limited PK data from patients with cirrhosis and esophageal varices indicate that its volume of distribution and elimination half-life are nearly twice the values measured in healthy individuals. The percentage of free etomidate in the plasma is markedly greater in patients with hepatic cirrhosis than in healthy controls [44.2 (2.1)% versus 24.9 (1.4)%, respectively] (Carlos et al., 1979). These findings highlight the necessity of cautious dose selection for patients with hepatic dysfunction. In the present study, ET-26-HCl at a dosage of 0.8 mg kg^-1^ did not significantly increase the safety risk in patients with mild to moderate hepatic impairment.

In addition, albumin levels and metabolic enzyme activity, among other parameters, are often considered less affected by mild to moderate liver impairment, but they can also be substantially compromised in patients with advanced liver impairment (Delcò et al., 2005; Verbeeck, 2008). Therefore, dosage adjustments may be necessary in patients with severe hepatic impairment (Child-Pugh C). However, a limitation of this study is that patients with severe hepatic impairment were not included due to safety concerns. The effect of severe hepatic impairment on ET-26-HCl remains unknown. Hence, the results of this study should not be extrapolated to patients with more severe liver dysfunction.

While our current study has enhanced the understanding of the PK, safety, and PD of ET-26-HCl in patients with mild to moderate hepatic impairment, it is important to acknowledge certain limitations. Given the small sample size, these negative findings remain inconclusive. Consequently, future studies with larger clinical sample sizes are required to corroborate the present results.

In conclusion, venous plasma ET-26-HCl exposure was ∼1.31- and ∼1.48-fold greater in patients with mild and moderate hepatic impairment, respectively, than in matched healthy controls. ET-26-HCl was generally safe and well tolerated by all participants in this study. Importantly, these findings should not be extrapolated to patients with severe hepatic impairment.

## Data Availability

The original contributions presented in the study are included in the article/Supplementary Material, further inquiries can be directed to the corresponding authors.
