# Supplementary material for: Pharmacokinetics, safety, and pharmacodynamics of intravenous methoxyethyl etomidate hydrochloride (ET-26-HCl) in Chinese participants with hepatic impairment
Source: Front Pharmacol. 2026 Jul 30;17:1827752. doi: 10.3389/fphar.2026.1827752 (PMC13469889; doi:10.3389/fphar.2026.1827752)
Supplement: Supplementary file 1 [file Supplementaryfile1.doc]

Supplementary information

Table S1 Pharmacokinetic characteristics of ETA in arterial and venous plasma

| Analyte | Parameters | Mild hepatic impairment (N=8) | Moderate hepatic impairment (N=8) | Normal hepatic function (N=8) |
| --- | --- | --- | --- | --- |
| ETA in arterial plasma | T_max_ (min) | 16.00(16.00,16.00) | 16.00(16.00,61.00) | 16.00(6.00,16.00) |
|  | C_max_ (ng mL^-1^) | 200(23.00) | 82.7(78.08) | 143(24.78) |
|  | AUC_0-t_ (ng·h mL^-1^) | 141(21.83) | 65.0(69.04) | 99.6(21.43) |
|  | MRT_0-t_ (h) | 0.51(2.62) | 0.53(5.49) | 0.49(3.83) |
|  | C_max,u_ (ng mL^-1^) | 63.5(20.88) | 30.9(65.91) | 43.2(28.28) |
|  | AUC_0-t,u_ (ng·h mL^-1^) | 45.1(21.11) | 24.3(57.98) | 30.2(24.95) |
| ETA in venous plasma | T_max_ (min) | 16.00(11.00,16.00) | 23.50(11.00,61.00) | 11.00(11.00,16.00) |
|  | C_max_ (ng mL^-1^) | 174(28.64) | 77.5(78.24) | 134(31.78) |
|  | AUC_0-t_ (ng·h mL^-1^) | 418(26.98) | 303(45.13) | 256(35.44) |
|  | AUC_0-∞_ (ng·h mL^-1^) | 450(25.32) | 340(41.14) | 273(35.43) |
|  | λ_z_(1/h) | 0.217(49.26) | 0.142(72.11) | 0.251(59.52) |
|  | t_1/2_ (h) | 3.19(49.26) | 4.90(72.11) | 2.76(59.52) |
|  | MRT_0-t_ (h) | 3.02(41.80) | 4.61(56.17) | 2.72(46.71) |
|  | MRT _0-∞_ (h) | 3.95(37.37) | 6.45(62.10) | 3.46(48.39) |
|  | AUC__%Extrap_ (%) | 6.62(43.84) | 9.34(61.91) | 6.25(10.51) |
|  | C_max,u_ (ng mL^-1^) | 55.4(27.55) | 29.0(67.78) | 40.5(34.14) |
|  | AUC_0-t,u_ (ng·h mL^-1^) | 133(29.34) | 113(43.37) | 77.6(39.41) |
|  | AUC_0-∞,u_ (ng·h mL^-1^) | 143(27.48) | 127(40.37) | 82.9(39.43) |
|  | Fu (%) | 31.92(7.31) | 37.74(14.66) | 30.34(3.91) |

The data are presented as the geometric means (CV%) except for T_max,_ which is presented as the median (range). C_max_: maximum observed concentration; AUC_0-t_: area under the curve from zero to last time of quantifiable concentration; AUC_0-∞_: area under the curve from zero to infinity; t_1/2_: terminal elimination half-life; T_max_: time to maximum concentration; MRT: mean residence time; fu: free fraction.

Table S2 Summary of adverse events

|  | Mild hepatic impairment (N=8), n (%) | Moderate hepatic impairment (N=8), n (%) | Normal hepatic function (N=8), n (%) | Total (N=24), n (%) |
| --- | --- | --- | --- | --- |
| All AEs | 6(75.0) | 6(75.0) | 5 (62.5) | 17 (70.8) |
| ADRs | 6(75.0) | 6(75.0) | 5 (62.5) | 17 (70.8) |
| SAEs | 0 | 0 | 0 | 0 |
| Sedative-related AEs | 0 | 0 | 3 (37.5) | 3 (12.5) |
| Cough | 1(12.5) | 1(12.5) | 2(25.0) | 4(16.7) |
| Elevated blood pressure | 4(50.0) | 0 | 0 | 4(16.7) |
| Involuntary muscle movement | 0 | 3(37.5) | 0 | 3(12.5) |
| Elevated blood bilirubin | 1(12.5) | 1(12.5) | 1(12.5) | 3(12.5) |
| Myoclonia | 1(12.5) | 0 | 1(12.5) | 2(8.3) |
| Respiratory depression | 0 | 0 | 2(25.0) | 2(8.3) |
| Decreased white blood cell count | 0 | 2(25.0) | 0 | 2(8.3) |
| Decreased neutrophil count | 0 | 2(25.0) | 0 | 2(8.3) |
| Decreased platelet count | 1(12.5) | 1(12.5) | 0 | 2(8.3) |
| Sleepiness | 0 | 1(12.5) | 0 | 1(4.2) |
| Dizziness | 0 | 1(12.5) | 0 | 1(4.2) |
| Bradycardia | 0 | 0 | 1(12.5) | 1(4.2) |
| Obstructive airway disease | 0 | 0 | 1(12.5) | 1(4.2) |
| Nausea | 1(12.5) | 0 | 0 | 1(4.2) |
| Gingival haemorrhage | 0 | 1(12.5) | 0 | 1(4.2) |
| Chest discomfort | 0 | 1(12.5) | 0 | 1(4.2) |
| Increased total bile acid | 0 | 1(12.5) | 0 | 1(4.2) |
| Elevated amylase | 0 | 0 | 1(12.5) | 1(4.2) |
| Positive urobilinogen | 1(12.5) | 0 | 0 | 1(4.2) |
| Aspartate aminotransferase | 1(12.5) | 0 | 0 | 1(4.2) |
| Decreased blood phosphorus | 0 | 1(12.5) | 0 | 1(4.2) |

AE, adverse events; ADR, adverse drug reaction; N, number of participants analyzed; n, number of participants who experienced at least one AE.

Table S3 Summary of the pharmacodynamics parameters

| Parameters | Mild hepatic impairment (N=8) | Moderate hepatic impairment (N=8) | Normal hepatic function (N=8) |
| --- | --- | --- | --- |
| BIS_peak_ | 62.6 ± 13.6  (39.0-78.0) | 48.4 ± 11.5  (37.0-68.0) | 50.3 ± 13.7  (38.0-75.0) |
| T_BISpeak_(min) | 4(2.00-6.00) | 4(2.00-9.00) | 4(0.00-7.00) |
| BIS AUC_0-t_ | 1100(20.99) | 1080(16.40) | 1300(9.89) |
| Fully alert time (min) | 10.309 ± 1.2774  (8.00-12.03) | 9.253 ± 1.0317  (8.00-10.00) | 10.020 ± 1.5328  (8.00-12.08) |

T_BISpeak_ is presented as the median (range); BIS AUC_0-t_ is presented as the geometric mean (CV%); BIS_peak_ and the fully alert time are presented as the means±SDs (range). BIS_peak_: BIS peak value (lowest BIS value); T_BISpeak_: time to BIS peak; BIS AUC_0-t_: area under the BIS curve from zero to the last collection time.

Table S4 The relationships between the C_max_ and the AUC of ET-26-HCl in arterial plasma and CYP2C19 gene subtypes

|  | PK parameters | CYP2C19 gene subtypes | N | F value | P value |
| --- | --- | --- | --- | --- | --- |
| Total ET-26-HCl | C_max_(ng/mL) | slow metabolizers | 2 | 1.241 | 0.309 |
|  |  | intermediate metabolizers | 12 |  |  |
|  |  | normal metabolizers | 10 |  |  |
|  | AUC_0-t_(h*ng/mL) | slow metabolizers | 2 | 0.480 | 0.625 |
|  |  | intermediate metabolizers | 12 |  |  |
|  |  | normal metabolizers | 10 |  |  |
|  | AUC_0-∞_(h*ng/mL) | slow metabolizers | 2 | 0.123 | 0.885 |
|  |  | intermediate metabolizers | 10 |  |  |
|  |  | normal metabolizers | 9 |  |  |
| Unbound ET-26-HCl | C_max,u_(ng/mL) | slow metabolizers | 2 | 1.923 | 0.171 |
|  |  | intermediate metabolizers | 12 |  |  |
|  |  | normal metabolizers | 10 |  |  |
|  | AUC_0-t,u_(h*ng/mL) | slow metabolizers | 2 | 1.032 | 0.374 |
|  |  | intermediate metabolizers | 12 |  |  |
|  |  | normal metabolizers | 10 |  |  |
|  | AUC_0-∞,u_(h*ng/mL) | slow metabolizers | 2 | 0.532 | 0.597 |
|  |  | intermediate metabolizers | 10 |  |  |
|  |  | normal metabolizers | 9 |  |  |

The CYP2C19 gene subtypes of the participants were tested in all the groups. Three gene subtypes are defined that indicate whether patients are normal metabolizers, intermediate metabolizers, or slow metabolizers, the ratio of which was n=10:12:2 in all the groups. Among them, normal hepatic function included 4 normal metabolism and 4 intermediate metabolism.; mild hepatic impairment comprised 4 normal metabolism, 3 intermediate metabolism, and 1 slow metabolism.; Moderate hepatic impairment consisted of normal metabolism, 5 intermediate metabolism, and 1 slow metabolism. This analysis is preliminary and limited by the small sample size, so no definitive conclusions can be drawn.

Table S5 The relationships between the C_max_ and the AUC of ET-26-HCl in venous plasma and CYP2C19 gene subtypes

|  | PK parameters | CYP2C19 gene subtypes | N | F value | P value |
| --- | --- | --- | --- | --- | --- |
| Total ET-26-HCl | C_max_(ng/mL) | slow metabolizers | 2 | 0.530 | 0.597 |
|  |  | intermediate metabolizers | 12 |  |  |
|  |  | normal metabolizers | 10 |  |  |
|  | AUC_0-t_(h*ng/mL) | slow metabolizers | 2 | 0.166 | 0.848 |
|  |  | intermediate metabolizers | 12 |  |  |
|  |  | normal metabolizers | 10 |  |  |
|  | AUC_0-∞_(h*ng/mL) | slow metabolizers | 2 | 0.174 | 0.842 |
|  |  | intermediate metabolizers | 10 |  |  |
|  |  | normal metabolizers | 9 |  |  |
| Unbound ET-26-HCl | C_max,u_(ng/mL) | slow metabolizers | 2 | 0.174 | 0.842 |
|  |  | intermediate metabolizers | 12 |  |  |
|  |  | normal metabolizers | 10 |  |  |
|  | AUC_0-t,u_(h*ng/mL) | slow metabolizers | 2 | 0.351 | 0.708 |
|  |  | intermediate metabolizers | 12 |  |  |
|  |  | normal metabolizers | 10 |  |  |
|  | AUC_0-∞,u_(h*ng/mL) | slow metabolizers | 2 | 0.351 | 0.708 |
|  |  | intermediate metabolizers | 10 |  |  |
|  |  | normal metabolizers | 9 |  |  |

The CYP2C19 gene subtypes of the participants were tested in all the groups. Three gene subtypes are defined that indicate whether patients are normal metabolizers, intermediate metabolizers, or slow metabolizers, the ratio of which was n=10:12:2 in all the groups. Among them, normal hepatic function included 4 normal metabolism and 4 intermediate metabolism.; mild hepatic impairment comprised 4 normal metabolism, 3 intermediate metabolism, and 1 slow metabolism.; Moderate hepatic impairment consisted of normal metabolism, 5 intermediate metabolism, and 1 slow metabolism. This analysis is preliminary and limited by the small sample size, so no definitive conclusions can be drawn.

Figure S1: Plasma concentration‒time curves for ET-26-HCl’s metabolite, ETA, on semilog scales. Left panel, arterial plasma graph (A); Right panel, venous plasma graph (B). The data are presented as the means ± standard deviations.

Figure S2: Forest plot of geometric mean ratios (90% confidence intervals, CIs) for BIS parameters in hepatic impairment groups versus the normal hepatic function group.The data are presented as the means ± standard deviations. BIS_peak_: BIS peak value (the lowest BIS value); BIS AUC_0-t_: area under the BIS curve from zero to last collection time; 90% CI: 90% confidence interval.Ratios (impaired/normal) are shown for BIS_peak_ and BIS AUC_0-t_. The vertical line at 100% represents no difference between groups. 90% CIs entirely within the 80-125% range (vertical red lines) indicate no clinically relevant difference.

Figure S3. Correlation scatterplots of the arterial ET-26-HCl exposure level (AUC_0-∞_) with the fully alert time, BISAUC_0-t_, BIS_peak_ and T_BISpeak_ for the normal hepatic function, mild hepatic impairment and moderate hepatic impairment groups.

Figure S4. Correlation scatterplots of the venous ET-26-HCl exposure level (AUC_0-∞_) with the fully alert time, BISAUC_0-t_, BIS_peak_ and T_BISpeak_ for the normal hepatic function, mild hepatic impairment and moderate hepatic impairment groups.

Figure S5. Correlation scatterplots of the ET-26-HCl C_max_ and AUC_0-t_ versus the severity of the hepatic impairment (mild [score 5–6] and moderate [score 7–9]). AUC_0-t_, area under the concentration‒time curve from the time of dosing to the last time point with measurable plasma concentrations of drug; C_max_, maximum observed plasma concentration.
